# Molecular detection of *Leishmania* spp. in road-killed wild mammals in the Central Western area of the State of São Paulo, Brazil

**DOI:** 10.1186/1678-9199-20-27

**Published:** 2014-06-16

**Authors:** Virginia Bodelão Richini-Pereira, Pamela Merlo Marson, Enio Yoshinori Hayasaka, Cassiano Victoria, Rodrigo Costa da Silva, Hélio Langoni

**Affiliations:** 1 Departamento de Higiene Veterinária e Saúde Pública, Faculdade de Medicina Veterinária e Zootecnia, Universidade Estadual Paulista (UNESP), Distrito de Rubião Júnior, s/n, Botucatu, SP, Brasil

**Keywords:** Road-killed animal, *Leishmania* spp, *Leishmania chagasi*, PCR, Zoonosis

## Abstract

**Background:**

Road-killed wild animals have been classified as sentinels for detecting such zoonotic pathogens as *Leishmania* spp., offering new opportunities for epidemiological studies of this infection.

**Methods:**

This study aimed to evaluate the presence of *Leishmania* spp. and *Leishmania chagasi* DNA by PCR in tissue samples (lung, liver, spleen, kidney, heart, mesenteric lymph node and adrenal gland) from 70 road-killed wild animals.

**Results:**

DNA was detected in tissues of one *Cavia aperea* (Brazilian guinea pig), five *Cerdocyon thous* (crab-eating fox), one *Dasypus septemcinctus* (seven-banded armadillo), two *Didelphis albiventris* (white-eared opossum), one *Hydrochoerus hydrochoeris* (capybara), two *Myrmecophaga tridactyla* (giant anteater), one *Procyon cancrivorus* (crab-eating raccoon), two *Sphiggurus spinosus* (porcupine) and one *Tamandua tetradactyla* (lesser anteater) from different locations in the Central Western part of São Paulo state. The *Leishmania chagasi* DNA were confirmed in mesenteric lymph node of one *Cerdocyon thous*. Results indicated common infection in wild animals.

**Conclusions:**

The approach employed herein proved useful for detecting the environmental occurrence of *Leishmania* spp. and *L. chagasi*, as well as determining natural wild reservoirs and contributing to understand the host-parasite interaction.

## Background

Leishmaniosis is a zoonotic, parasitic disease caused by kinetoplastid flagellate protozoan parasites of the genus *Leishmania* that infects several mammal species, including humans, and is transmitted by the phlebotomine sandfly. *Leishmania* species include visceral, cutaneous and mucocutaneous forms of the disease in both the Old and New Worlds [1,2].

Great concern has been sparked by the contribution that global warming might be making to the recent increase in the number of reported cases and geographical areas [3]. Environmental, demographic and human behavioral factors contribute to the changing landscape of leishmaniasis, which includes increased risk factors for zoonotic cutaneous leishmaniasis and new scenarios associated with the zoonotic visceral leishmaniasis [4].

Studies on *Leishmania* spp. in wild animals have become more numerous in Brazil due to the importance of these species in the life cycle of leishmaniasis [5]. Studies involving road-killed instead of laboratory research animals have become more frequent in helminthological, epidemiological, morphological and genetic areas [6-12]. However, the use of molecular techniques for detection of microorganisms in these samples is recent [13-17].

Despite the difficulty of culturing and histopathologically analyzing tissue samples from road-killed animals, molecular techniques can be used in the identification and typing of pathogens by polymerase chain reaction (PCR), which presents high specificity and sensitivity to a certain fragment of the pathogen’s specific DNA [18].

Leishmania kinetoplastic DNA (kDNA)-specific probes have been used for the detection and identification of this protozoan, and demonstrated to be useful for epidemiological field studies because a large number of samples can be handled simultaneously [19]. A minicircle of kDNA (0.8 to 1 kb in length) is an ideal target, since it is present in 10,000 copies per cell and because its sequences are known for most *Leishmania* species [20].

The present work aimed to describe possible new hosts for leishmaniasis by using molecular tools to detect *Leishmania* spp. in tissues of road-killed wild mammals. Thus, research into new hosts by molecular techniques is distinctive in epidemiological studies of pathogens and represents suitable indicators of environmental contamination by *Leishmania* spp. [21].

## Methods

### Animals and studied area

Seventy road-killed wild animals were studied: one *Callithrix penicillata* (black-tufted marmoset), four *Cavia aperea* (Brazilian guinea pig), one *Cebus apella* (capuchin monkey), 13 *Cerdocyon thous* (crab-eating fox), three *Dasypus novemcinctus* (nine-banded armadillo), one *Dasypus septemcinctus* (seven-banded armadillo)*,* nine *Didelphis albiventris* (white-eared opossum), one *Eira barbara* (tayra), one *Euphractus sexcinctus* (six-banded armadillo)*,* two *Gallictis vittata* (grison), two *Hydrochoerus hydrochaeris* (capybara), one *Leopardus tigrinus* (leopard cat), five *Lepus europaeus* (brown hare), three *Lutreolina crassicaudata* (latrine opossum), two *Mazama gouazoubira* (brown brocket deer), one *Myocastor coypus* (coypu), six *Myrmecophaga tridactyla* (giant anteater), three *Procyon cancrivorus* (crab-eating raccoon), two *Puma concolor* (cougar), two *Rattus rattus* (black rat), five *Sphiggurus spinosus* (porcupine) and two *Tamandua tetradactyla* (lesser anteater). Only recently killed animals (1–7 hours) and those with no exposed viscera were collected. This study is in accordance with the Brazilian Institute of Environment and Renewable Natural Resources’ (IBAMA) normative statement n. 119 of October 11, 2006, chapter VI, art .26, which authorizes the collection and transport of animals that were found dead for scientific or didactic purposes. This work was also approved by the Ethics Committee for Animal Experimentation at our Institution (CEEA/FMVZ n.211/2008).

The geographic positions of the road-killed animals, established through global positioning system (GPS), were plotted on a digital map using a geographic database by the TerraView 3.6.0 [22].

### Molecular detection

DNA extraction from the animals’ tissue samples (lung, spleen, liver, kidney, heart, mesenteric lymph node and adrenal gland) was carried out by using the kit Illustra™ Tissue & Cells Genomic Prep Mini Spin (GE Healthcare, USA). PCR reactions were performed by employing the primers LinR4 (5’-GGGTTGGTGTAAAATAGGG-3’) and Lin19 (5’-CAGAACGCCCCTACCCG-3’), described by Aransay *et al*. [20], to amplify a 720 bp fragment. Samples positive for *Leishmania* spp. PCR were also assayed for *Leishmania braziliensis* complex and *Leishmania mexicana* complex [23,24]. Genus-specific primers for *Leishmania* spp. were used in order to identify the DNA of all possible *Leishmania* species that cause visceral or cutaneous leishmaniasis. The cycling profile consisted of an initial denaturation at 95°C for three minutes, followed by 30 cycles at 95°C for 30 seconds, 63°C for 30 seconds and 72°C for one minute, and a final extension at 72°C for seven minutes. Positive controls were included in each assay and consisted of 10 ng of DNA extracted from *Leishmania major* (MHOM/SU/1973/5-ASKH) and *L. chagasi* (MHOM/BR/2002/LPC-RPV). Negative controls were: ultrapure water and DNA from *T. cruzi* (ColTryp 0032/MCAN/BR/2008/CAO) that were added to the mix-PCR. The PCR mixture was composed of 10 mM Tris HCl pH 8.0, 50 mM KCl, 1.5 mM MgCl_2_, 0.2 mM dNTP, 10 ρmol of each primer, 0.2 units of *Taq* DNA polymerase, and 10 ng DNA template.

The amplification of *Leishmania chagasi* DNA was performed utilizing primers Lc14 (5’-CGCACGTTATATCTACAGGTTGAG-3’) and Lc15 (5’- TGTTTGGGATTGAGGTAATAGTGA-3’) on a 190 bp fragment, by using the following cycling profile: initial denaturation at 94°C for four minutes, 40 cycles of 94°C for 30 seconds, 59°C for 30 seconds, 72°C for 30 seconds, and 70°C for ten minutes. Positive controls were included in each assay and consisted of 10 ng of DNA extracted from *L. chagasi* (MHOM/BR/2002/LPC-RPV). Negative controls were: ultrapure water and DNA from *T. cruzi* (ColTryp 0032/MCAN/BR/2008/CAO) that were added to the mix-PCR. The PCR mixture was composed of 10 mM Tris HCl pH 8.0, 50 mM KCl, 1.5 mM MgCl_2_, 0.2 mM dNTP, 10 ρmol of each primer, 0.5 units of *Taq* DNA polymerase, and 10 ng DNA template.

Amplification was performed in a MasterCycler EP gradient (Eppendorf, USA). The sequence was analyzed by electrophoresis in 1.5% agarose with SYBR® safe DNA gel stained (Invitrogen, USA), and visualized in an image analyzer (GelDoc-It™ Imaging System – UVP, USA) by using VisonWorks®LS Software. Amplicons were purified by using ExoSap (USB, USA) and the sequencing reactions were carried out on both strands in a 3500 Genetic Analyzer (Applied Biosystems). The obtained sense and antisense sequences were visualized (Chromas 2.3 software, Technelysium Pty Ltd, Australia), aligned by the software MEGA 4 and compared with the NCBI database using BLASTn (Basic Local Alignment Tool for Nucleotide) [25].

## Results and discussion

The present results draw attention to a very important source of research and emphasize the importance of using this biological resource in an epidemiological study of zoonotic infection.

One of the main problems in elucidating leishmaniasis epidemiology is to identify and confirm that a vertebrate host is a natural reservoir. The natural reservoirs are widely unknown because of the difficulties in capturing a sufficient number of wild animals and due to the techniques used in isolating and identifying the parasite.

This approach of using road-killed wild animals for the molecular detection of *Leishmania* spp. may represent a useful alternative to the utilization of captured ones in research studies, as indicated by animal research ethics committees. In the present study, a great diversity of road-killed wild mammal species was found. Culture analysis and histopathology are difficult and laborious. Sensitive and specific molecular tools allow pathogens to be identified without the need of culturing.

In this paper, molecular detection of *Leishmania* spp. and *Leishmania chagasi* was attempted from several wildlife species using PCR. Several studies have reported the presence of this parasite in mammalian species, including rodents, carnivores, primates and marsupials [5,26-29].

**Figure 1:**
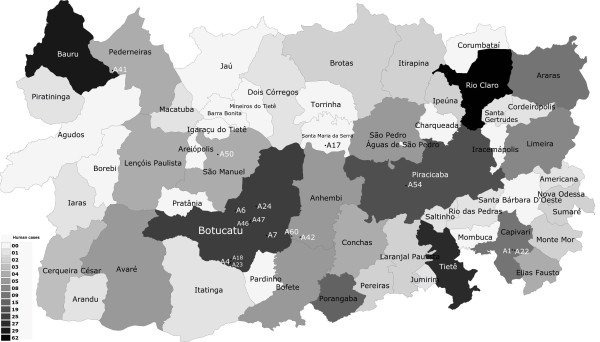
**Geographic location of road-killed animals employed for *Leishmania *** **spp. molecular detection, correlating to the occurrence of cases of human cutaneous leishmaniasis.**

**Figure 2:**
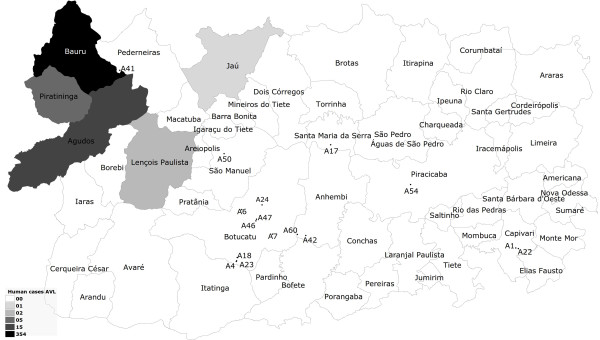
**Geographic location of the road-killed animals employed for *Leishmania *** **spp. molecular detection, correlating to the occurrence of cases of human visceral leishmaniasis.**

Table 1 contains the results of the PCR and identifies percentages of amplicon obtained in road-killed wild animals positive for *Leishmania* spp. and *Leishmania chagasi* from deposited homologue DNA sequences, as determined by BLASTn analysis. Figure 1 illustrates the human cutaneous leishmaniasis data corresponding to the cases seen in the central western area of the state of São Paulo, Brazil, from 1998 to 2010 [30]. Figure 2 displays the human visceral leishmaniasis data corresponding to the cases seen in the central western area of the state of São Paulo, Brazil, from 1998 to 2010 and geographic location of the positive road-killed animals evaluated [31].

**Table 1:** **Data on road-killed wild animals, including the sex, tissue, PCR and sequencing results for molecular detection on *Leishmania *** **spp. and *Leishmania chagasi***

**Species**	**Animal**	**Sex**	**Tissue (PCR positive)**	**% identity/GenBank access**
*Procyon cancrivorus*	A1	Male	kidney	99%/AJ270142.1 *Leishmania* spp.
*Cerdocyon thous*	A4	Male	heart, mesenteric lymph node	100%/AJ270142.1 *Leishmania* spp.
*Cerdocyon thous*	A6	Male	spleen, heart	100%/AJ270142.1 *Leishmania* spp.
*Cerdocyon thous*	A7	Male	heart	100%/AJ270141.1 *Leishmania* spp.
*Cavia aperea*	A17	Male	heart	99%/AJ270141.1 *Leishmania* spp.
*Dasypus septemcinctus*	A18	Male	liver	100%/AJ270141.1 *Leishmania* spp.
*Sphiggurus spinosus*	A22	#	liver, spleen	100%/AJ270142.1 *Leishmania* spp.
*Tamandua tetradactyla*	A23	Male	lung, liver, mesenteric lymph node	100%/AJ270142.1 *Leishmania* spp.
*Shiggurus spinosus*	A24	Female	spleen, kidney, heart	100%/AJ270142.1 *Leishmania* spp.
*Cerdocyon thous*	A41	Male	liver, mesenteric lymph node	100%/AF308682.1 *Leishmania chagasi*
*Myrmecophaga. tridactyla*	A42	Male	lung, kidney, heart, mesenteric lymph node	100%/AJ270142.1 *Leishmania* spp.
*Didelphis albiventris*	A46	Male	liver, spleen, kidney	100%/AJ270142.1 *Leishmania* spp.
*Didelphis albiventris*	A47	Male	lung	100%/AJ270142.1 *Leishmania* spp.
*Hydrochoerus hydrochaeris*	A50	Female	lung	100%/AJ270142.1 *Leishmania* spp.
*Cerdocyon thous*	A54	Male	lung, spleen	100%/AJ270142.1 *Leishmania* spp.
*Myrmecophaga tridactyla*	A60	Male	lung	100%/AJ270142.1 *Leishmania* spp.

#not available.

Leishmania DNA was detected in 5/12 (41.67%; CI95% 19.22-68.42%) samples from *Cerdocyon thous* (crab-eating fox). A previous report indicates seropositivity in wild non-captive *Cerdocyon thous*[5,28]. The importance of these animals as reservoirs depends on their ability to transmit the infection to sandflies rather than on their infection rate; it is also a function of their capability to (re)introduce the pathogen into *Leishmania*-free dog populations [32].

In the current study, *Leishmania* spp. DNA was detected in 1/3 *Procyon cancrivorus* (crab-eating raccoon). Voltarelli *et al.*[33] reported the presence of *Leishmania* antibodies in *Procyon cancrivorus* in Northwestern Paraná. These findings suggest that these species can act as a reservoir for *Leishmania* spp.

The members of Didelphidae, represented by *Didelphis albiventris* specimens (white-eared opossums), are habitat generalists and currently occur in areas near dwellings, including farms, yards and urban centers [34]. This species is already proven to be a leishmaniasis reservoir and, for its synanthropic habits, it plays an important role in the peridomestic-forest traffic of degraded areas [35-37]. The present study confirms that molecular detection of *Leishmania* spp. in 2/8 group members may be common, as was already described in several regions of Brazil: Manaus, Amazonas state; in Barra de Guarituba, Rio de Janeiro state; in Amaraji, Pernambuco; and Bauru, São Paulo state [38-41].

Rodents were represented by five *S. spinosus* (porcupine), four *C. aperea* (Brazilian guinea pig), two *Rattus rattus* (black rat), two *Hydrochoerus hydrochaeris* (capybara) and one *M. coypus* (coypu). Current data show that 8/14 (57.14%; CI95% 35.14-82.34%) specimens of wild rodents were positive, a finding that corroborates the literature that considered some rodent as reservoirs of *Leishmania* spp*.*

The superorder Xenarthra was represented by 13 specimens: three *D. novemcinctus* (nine-banded armadillo), one *E. sexcinctus* (six-banded armadillo), one *D. septemcinctus* (seven-banded armadillo), two *T. tetradactyla* (lesser anteater) and six *M. tridactyla* (giant anteater). These animals present some peculiar physiological and ecological characteristics including a weak immune system and low body temperature, besides the fact that they live literally immersed in soil and organic matter, mainly in tropical and subtropical regions, under biotic and abiotic conditions that promote multiple encounters with a diverse group of pathogens and vectors.

The present study confirms the occurrence of *Leishmania* spp. DNA in armadillos (one *D. septemcintus*) and anteaters (one *T. tetradactyla* and two *M. tridactyla*). Casadeval and Pirofski [42] clarified many points on virulence and pathogenicity regarding host immune response and pathogen activity. According to the authors, there are classes of pathogenic microorganisms varying from those that provoke damage in hosts that present an extremely weak immune response to others that cause disease only in a situation of very strong immune response. Therefore, it seems reasonable to consider *Leishmania* spp. to be a pathogen whose ability to provoke disease also depends on host immune response. Since the cellular immune response is weak in armadillos and anteaters, it is possible to detect yeast cells in many of their organs; however, this is not sufficient to cause disease as observed in human hosts. Taken together, these factors make xenarthrans suitable models for studying host-pathogen interaction [43].

These animals are assumed to be sources of infection since the agent’s DNA was found in internal organs; in addition, parasitism may occur in internal and cutaneous organs, facilitating transmission from the blood meal by the vector that inoculates promastigote forms of the agent into the man while sucking.

The identities of the amplicon were confirmed by direct double-strand sequencing which showed 100% similarity with *L. chagasi* sequence deposited at GenBank (access number AF308682.1) (Table 1).

Even without the DNA detection of the cutaneous leishmaniasis agents, the positive results for *Leishmania* spp. are interesting. Of the 20 species described in the New World, five have never been reported to have caused visceral human leishmaniasis: *Leishmania enriettii, Leishmania hertigi*, *Leishmania deanei, Leishmania aristidesi* and *Leishmania forattinii*[44-48]. The *L. forattinii* was isolated from pooled liver and spleen of opossum *Didelphis marsupialis* captured in Conchas, SP, Brazil [48,49].

It is suggested that the species is *Leishmania forattinii* and that the evaluated site is close to that where the parasite was first isolated, since the species nucleotide sequence deposited at the GenBank was not found. Considering the occurrence of both the cutaneous and visceral form, in the studied municipalities, it must be emphasized that the sandfly vector may be present and serve as transmitter of *Leishmania* to these animals and humans.

These findings corroborate the worldwide distribution of *Leishmania* spp., considering the wide variety of intermediate hosts that contribute to the epidemiological transmission chain of this infection.

It is important to emphasize that Bauru, SP, is endemic for leishmaniasis; therefore, our results indicate the need for epidemiological molecular biology research on environmental contamination by *L. chagasi.*

It was possible to evaluate 22 different wild species, without the necessity of exerting a laborious sampling effort. In fact, the numbers and diversity of road-killed animals are considerably higher and, in general, they are killed after their own natural habitats had been invaded by roads [50]. In this manner, the geographic coordinates of the locations of the infected animals are well-integrated in databases that use the geographical information systems (GIS), thus contributing to a better understanding of pathogen distribution.

These results show risk factors such as free movement of the circulating parasite and vectors, as well as the importance of road-killed animals as possible reservoirs for the transmission of *Leishmania* spp. in addition to the significance of the environment and ecology of these positive mammals in the interaction of Leishmania with different *Leishmania* species that may be pathogenic to humans.

## Conclusions

The presented results focus that road-killed animals may serve as an important reservoir for transmission of *Leishmania* spp. and *L. chagasi*, as well as contributing to understand the host-parasite interaction.

## Competing interests

The authors declare that there are no competing interests.

## Authors’ contributions

VBRP participated in the design of the study, data collection, laboratory tests, analysis and interpretation of data, writing and editing of the manuscript. PMM and EYH participated in the data collection, laboratory tests and took part in the writing. CV participated in geographical location of animals, analysis and interpretation of data, writing and revision of the manuscript. RCS participated in the analysis and interpretation of data, writing and revision. HL was responsible for the coordination, study design, analysis and interpretation of data, writing and editing of the manuscript. All authors read and approved the final manuscript.

## References

[B1] Gontijo B Carvalho MLR Leishmaniose tegumentar americana Rev Soc Bras Med Trop 2003 36 1 71 80 12715066 10.1590/s0037-86822003000100011

[B2] Gontijo CMF Melo MN Leishmaniose visceral no Brazil: quadro atual, desafios e perspectivas Rev Bras Epidemiol 2004 7 3 338 349 10.1590/S1415-790X2004000300011

[B3] Desjeux P Leishmaniasis: currents situation and new perspectives Comp Immunol Microbiol Infect Dis 2004 27 5 305 318 10.1016/j.cimid.2004.03.004 15225981

[B4] Gramiccia M Gradoni L The current status of zoonotic leishmaniases and approaches to disease control Int J Parasitol 2005 35 11–12 1169 1180 16162348 10.1016/j.ijpara.2005.07.001

[B5] Luppi MM Malta MC Silva TM Silva FL Motta RO Miranda I Ecco R Santos RL Visceral leishmaniasis in captive wild canids in Brazil Vet Parasitol 2008 155 1–2 146 151 18556130 10.1016/j.vetpar.2008.04.024

[B6] Coyner DF Wooding JB Forrester DJ A comparison of parasitic helminths and arthropods from two subspecies of fox squirrels (*Sciurus niger*) in Florida J Wildl Dis 1996 32 3 492 497 10.7589/0090-3558-32.3.492 8827675

[B7] Cheadle MA Tanhauser SM Dame JB Sellon DC Hines M Ginn PE Mackay RJ Greiner EC The nine-banded armadillo (*Dasypus novemcinctus*) is an intermediate host for *Sarcocystis neurona* Int J Parasitol 2001 31 4 330 335 10.1016/S0020-7519(01)00177-1 11306111

[B8] Foster GW Main MB Kinsella JM Dixon LM Terrell SP Forrester DJ Parasitic helminths and arthropods of coyotes (*Canis latrans*) from Florida USA Comp Parasitol 2003 70 2 162 166 10.1654/4081

[B9] Nelder MP Reeves WK Ectoparasites of road-killed vertebrates in northwestern South Carolina, USA Vet Parasitol 2005 129 3–4 313 322 15845287 10.1016/j.vetpar.2004.02.029

[B10] Ferroglio E Ragagli C Trisciuoglio A Physaloptera sibirica in foxes and badgers from the Western Alps (Italy) Vet Parasitol 2009 163 1–2 164 166 19411141 10.1016/j.vetpar.2009.04.005

[B11] Hoppe EG Araújo de Lima RC Tebaldi JH Athayde AC Nascimento AA Helminthological records of six-banded armadillos *Euphractus sexcinctus* (Linnaeus, 1758) from the Brazilian semi-arid region, Patos county, Paraíba state, including new morphological data on *Trichohelix tuberculata* (Parona and Stossich, 1901) Ortlepp, 1922 and proposal of *Hadrostrongylus ransomi* nov. comb Braz J Biol 2009 69 2 423 428 10.1590/S1519-69842009000200027 19675948

[B12] Miquel J Foronda P Torres J Swiderski Z Feliu C Ultrastructural study of the spermatozoon of *Taenia taeniaeformis* (Batsch, 1786) (Cestoda, Cyclophyllidea, Taeniidae), an intestinal parasite of *Felis catus* from La Palma (Canary Islands, Spain) Parasitol Res 2009 104 6 1477 1483 10.1007/s00436-009-1351-y 19205741

[B13] Richini-Pereira VB Bosco SMG Griese J Theodoro RC Macoris SA da Silva RJ Barrozo L Tavares PM Zancopé-Oliveira RM Bagagli E Molecular detection of *Paracoccidioides brasiliensis* in road-killed wild animals Med Mycol 2008 46 1 35 40 10.1080/13693780701553002 17885959

[B14] Richini-Pereira VB Bosco SMG Theodoro RC Barrozo L Pedrini SCB Rosa PS Bagagli E Importance of xenarthrans in the eco-epidemiology of *Paracoccidioides brasiliensis* BMC Res Notes 2009 2 1 6 10.1186/1756-0500-2-1 19919716 PMC2784786

[B15] Richini-Pereira VB Bosco SMG Theodoro RC Barrozo L Bagagli E Road-killed wild animals: a preservation problem useful for eco-epidemiological studies of pathogens J Venom Anim Toxins incl Trop Dis 2010 16 4 607613 http://www.scielo.br/scielo.php?script=sci_arttext&pid=S1678-91992010000400011

[B16] Zhao C Onuma M Asakawa M Nagamine T Kuwana T Preliminary studies on developing a nested PCR assay for molecular diagnosis and identification of nematode (*Heterakis isolonche*) and trematode (*Glaphyrostomum* sp.) in Okinawa rail (*Gallirallus okinawae*) Vet Parasitol 2009 163 1–2 156 160 19394146 10.1016/j.vetpar.2009.03.038

[B17] Pedrini SC Rosa PS Medri IM Mourão G Bagagli E Lopes CA Search for *Mycobacterium leprae* in wild mammals Braz J Infect Dis 2010 14 1 47 53 10.1016/S1413-8670(10)70010-6 20428654

[B18] Persing DH Smith TF Tenover FC White TJ Diagnostic Molecular Microbiology: Principles and Applications 1993 Washington, D.C: American Society for Microbiology 423 430

[B19] Esseghir SA Ftaiti A Ready PB Khdraoui B Zaafouri K Dellagi K Ben Ismail R The squash blot technique and the detection of *Leishmania major* in *Phlebotomus papatasi* in Tunisia Arch Inst Pasteur Tunis 1993 70 3–4 493 496 7802506

[B20] Aransay AM Scoulica E Tselentis Y Detection and identification of *Leishmania* DNA within naturally infected sand flies by seminested PCR on minicircle kinetoplastic DNA Appl Environ Microbiol 2000 66 55 1933 1938 10788363 10.1128/aem.66.5.1933-1938.2000 PMC101436

[B21] Gennari SM Canón-Franco WA Yai LE de Souza SL Santos LC Farias NA Ruas J Rossi FW Gomes AB Seroprevalence of *Toxoplasma gondii* antibodies from wild canids from Brazil Vet Parasitol 2004 121 3–4 337 340 15135875 10.1016/j.vetpar.2004.02.023

[B22] INPE Instituto Nacional de Pesquisas Espaciais TerraView: Software v.3.6.0 [http://www.dpi.inpe.br/terraview/index.php]

[B23] de Bruijn MH Barker DC Diagnosis of New World leishmaniasis: specific detection of species of the *Leishmania braziliensis* complex by amplification of kinetoplast DNA Acta Trop 1992 52 1 45 58 10.1016/0001-706X(92)90006-J 1359760

[B24] Eresh S McCallum SM Barker DC Identification and diagnosis of *Leishmania mexicana* complex isolates by polymerase chain reaction Parasitololy 1994 109 Pt 4 423 433 10.1017/s0031182000080677 7800410

[B25] Tamura K Dudley J Nei M Kumar S MEGA4: Molecular Evolutionary Genetics Analysis (MEGA) software version 4.0 Mol Biol Evol 2007 24 8 1596 1599 10.1093/molbev/msm092 17488738

[B26] Alexander B Lozano C Barker DC McCann SH Adler G Detection of *Leishmania* (*Viannia*) *braziliensis* complex in wild mammals from Colombian coffee plantations by PCR and DNA hybridization Acta Trop 1998 69 1 41 50 10.1016/S0001-706X(97)00114-9 9588240

[B27] Travi BL Osorio Y Becerra MT Adler GH Dynamics of *Leishmania chagasi* infection in small mammals of the undisturbed and degraded tropical dry forests of northern Colombia Trans R Soc Trop Med Hyg 1998 92 3 275 278 10.1016/S0035-9203(98)91009-4 9861395

[B28] Curi NHA Miranda I Talamoni AS Serologic evidence of *Leishmania* infection in free-ranging wild and domestic canids around a Brazilian National Park Mem Inst Oswaldo Cruz 2006 101 1 99 101 10.1590/S0074-02762006000100019 16699717

[B29] Papadogiannakis E Spanakos G Kontos V Menounos PG Tegos N Vakalis N Molecular detection of *Leishmania infantum* in wild rodents (*Rattus norvegicus*) in Greece Zoonoses Public Health 2010 57 7–8 23 25 19912600 10.1111/j.1863-2378.2009.01264.x

[B30] Centro de Vigilância Epidemiológica Leishmaniose tegumentar americana. Distribuição do número de casos de Leishmaniose Tegumentar por município provável de infecção. Estado de São Paulo, 1998–2012 http://www.cve.saude.sp.gov.br/htm/zoo/lta_lpi.htm

[B31] Centro de Vigilância Epidemiológica Leishmaniose visceral americana. Distribuição do número de casos e óbitos de LVA segundo município e GVE de infecção. Estado de São Paulo 1998–2011 http://www.cve.saude.sp.gov.br/htm/zoo/lvah_lpi.htm

[B32] Dressen DW *Toxoplasma gondii* infections in wildlife J Am Vet Med Assoc 1990 196 274 276 2404927

[B33] Voltarelli EM Arraes SMAA Perles TF Lonardoni MVC Teodoro U Silveira TGV Serological survey for *Leishmania* sp. infection in wild animals from the municipality of Maringá, Paraná State, Brazil J Venom Anim Toxins incl Trop Dis 2009 15 4 732 744 http://www.scielo.br/scielo.php?script=sci_arttext&pid=S1678-91992009000400011 10.1590/S1678-91992009000400011

[B34] Câmara T Murta R Mamíferos da Serra do Cipó 2003 PUC, Minas, Museu de Ciências Naturais: Belo Horizonte

[B35] Forrester DJ Parasites and Diseases of Wild Mammals in Florida 1992 First Gainesville: University Press of Florida 459

[B36] Guerra JAO Ribeiro JAS Coelho LIARC Barbosa MGV Paes MG Epidemiologia da leishmaniose tegumentar na comunidade São João, Manaus, Amazonas Brasil Cad Saúde Pública 2006 22 11 2319 2327 10.1590/s0102-311x2006001100006 17091169

[B37] Quintal AP Ribeiro Ede S Rodrigues FP Rocha FS Floeter-Winter LM Nunes CM *Leishmania* spp. in *Didelphis albiventris* and *Micoureus paraguayanus* (Didelphimorphia: Didelphidae) of Brazil Vet Parasitol 2011 176 2–3 112 119 21144664 10.1016/j.vetpar.2010.11.011

[B38] Arias JR Naiff RD The principal reservoir host of cutaneous leishmaniasis in the urban areas of Manaus, Central Amazon of Brazil Mem Inst Oswaldo Cruz 1981 76 3 279 286 7348777 10.1590/s0074-02761981000300005

[B39] Cabrera MAA Paula AA Camacho LAB Marzochi MCA Xavier SC da Silva AVM Jansen AM Canine visceral leishmaniasis in Barra de Guaratiba, Rio de Janeiro, Brazil: assessment of risk factors Rev Inst Med Trop Sao Paulo 2003 45 2 79 83 10.1590/S0036-46652003000200005 12754572

[B40] Brandão-Filho SP Brito ME Carvalho FG Ishikawa EA Cupolillo E Floeter-Winter L Shaw JJ Wild and synanthropic host of *Leishmania* (Viannia) *braziliensis* in the endemic cutaneous leismaniasis locality of Amaraji, Pernambuco State, Brazil Trans R Soc Trop Med Hyg 2003 97 3 291 296 10.1016/S0035-9203(03)90146-5 15228244

[B41] Santiago ME Vasconcelos RO Fattori KR Munari DP Michelin Ade F Lima VM An investigation of *Leishmania* spp. in *Didelphis* spp. from urban and peri-urban areas in Bauru (São Paulo, Brazil) Vet Parasitol 2007 150 4 283 290 10.1016/j.vetpar.2007.09.026 17996372

[B42] Casadevall A Pirofski LA Host-pathogen interactions: redefining the basic concepts of virulence and pathogenicity Infect Immun 1999 67 8 3703 3713 10417127 10.1128/iai.67.8.3703-3713.1999 PMC96643

[B43] Bagagli E Bosco SMG Viscaino SF, Loughry WJ Armadillos and dimorphic pathogenic fungi: ecological and evolutionary aspects The Biology of the Xenarthra 2008 First Gainesville: University Press of Florida 103 110

[B44] Muniz J Medina HSG Leishmaniose tegumentar do cobaio *(Leishmania enriettii)* Arq Biol Tecnol 1948 3 2 7 25 18908199

[B45] Herrer A *Leishmania hertigi* sp. n., from the tropical porcupine, *Coendou rothschildi Thomas* J Parasitol 1971 57 3 626 629 10.2307/3277928 5090970

[B46] Lainson R Shaw JJ Leishmanias of neotropical porcupines: *Leishmania hertigi deanei* nov. subsp Acta Amaz 1977 7 1 51 57

[B47] Lainson R Shaw JJ Lumsden WHR, Evans DA The role of animals in the epidemiology of South American leishmaniasis The Biology of the Kinetoplastida, Volume 2 1979 London, New York, San Francisco: Academic Press 1 116

[B48] Yoshida ELA Silva R Cortez LS Corrêa FMA Encontro de espécie do gênero *Leishmania* em *Didelphis marsupialis aurita* no Estado de São Paulo, Brasil Rev Inst Med Trop Sao Paulo 1979 21 110 113 573492

[B49] Yoshida ELA Cuba CA Pacheco RS Cupolillo E Tavares CC Machado GMC Momen H Grimaldi JG Description of *Leishmania (Leishmania) forattinii* sp. n., a new parasite infecting opossums and rodents in Brazil Mem Inst Oswaldo Cruz 1993 88 3 397 406 10.1590/S0074-02761993000300008

[B50] Laurance WF Goosem M Laurance SG Impacts of roads and linear clearings on tropical forests Trends Ecol Evol 2009 24 12 659 669 10.1016/j.tree.2009.06.009 19748151

